# Bridging the (Brexit) divide: Effects of a brief befriending meditation on affective polarization

**DOI:** 10.1371/journal.pone.0267493

**Published:** 2022-05-11

**Authors:** Otto Simonsson, Simon B. Goldberg, Joseph Marks, Liuxin Yan, Jayanth Narayanan

**Affiliations:** 1 Department of Clinical Neuroscience, Center for Psychiatry Research, Karolinska Institutet, Stockholm, Sweden; 2 Department of Sociology, University of Oxford, Oxford, United Kingdom; 3 Center for Healthy Minds, University of Wisconsin—Madison, Madison, WI, United States of America; 4 Department of Counseling Psychology, University of Wisconsin—Madison, Madison, WI, United States of America; 5 Department of Experimental Psychology, University College London, London, United Kingdom; 6 NUS Business School, National University of Singapore, Singapore, Singapore; University of Edinburgh, UNITED KINGDOM

## Abstract

The European Union Brexit referendum has divided the British electorate, with high levels of animosity between those who affiliate with the Remain side (Remainers) and the Leave side (Leavers) of the debate. Previous research has shown that a brief befriending meditation reduces affective polarization among Democrats and Republicans in the United States, but the results have not been replicated in a non-US sample and the psychological mechanisms underlying the effects have yet to be examined. The present study therefore used a post-test only randomized controlled design to investigate the effects of a brief befriending meditation on affective polarization among Remainers and Leavers (n = 922). Results showed that participants in the befriending condition scored modestly lower on affective polarization than participants in the attentional control condition (t(921) = 2.17, p = .030, d = 0.14) and that perceived commonality with the political outgroup mediated the effects. In sum, audio-guided befriending practices may be a highly scalable means to reduce high levels of affective polarization through increasing perceived commonality.

## Introduction

On the 23^rd^ June 2016, the British electorate was asked in a nationwide referendum whether the United Kingdom (UK) should remain a member of the European Union (EU) or leave. The results showed that 51.9% of the votes were cast in favor of leaving the EU (Brexit), which eventually led the UK to formally leave the EU [1]. While the question of EU membership had not been a salient issue to British voters before the referendum, Brexit identities (i.e., Remainers and Leavers) quickly became a central part of British politics, with longitudinal surveys showing that British adults had stronger emotional attachment for their Brexit identity than their party identity [2].

The emergence of Brexit identities has been accompanied by strong ingroup favoritism bias between the two sides in the debate. For example, Remainers and Leavers have been shown, at the aggregate level, to attribute more positive traits to their own side and more negative traits to the other side–a phenomenon known as affective polarization [2]. While relatively little is known about the consequences of affective polarization in the British political context, evidence from the United States suggests that high levels of affective polarization may have potentially harmful consequences for society [3–8]. It is therefore important to investigate scalable interventions that can reduce it.

Previous studies have found several ways to reduce affective polarization [7,9,10]. For instance, a recent study found that simply imagining positive interactions with the political outgroup reduced partisan animosity among Democrats and Republicans [11], which corresponds with imagined intergroup contact effects on intergroup attitudes more generally [12,13]. It might be difficult to scale such an intervention in a polarized real-world setting, however, especially as people with high levels of affective polarization may be reluctant to voluntarily imagine positive political outgroup interactions.

An alternative approach could be to use a similar intervention that does not make explicit mention of the political outgroup and still targets the same underlying mechanism as imagined intergroup contact. Prior findings suggest that a key mechanisms underlying imagined intergroup contact effects on affective polarization may be perceived commonality between the self and the political outgroup [14]. While several interventions have been shown to increase perceived commonality in an intergroup context [15,16], lovingkindness- and compassion-based practices such as befriending meditation could represent a more suitable method for reducing affective polarization, given the potential barriers associated with the imagined intergroup contact approach.

Both befriending meditation and imagined intergroup contact involves bringing different types of people to mind, but befriending meditation does not make any explicit mention of political groups and might therefore be more accessible and scalable than the imagined intergroup contact approach, especially given the widespread popularity of meditation practices and meditation-based apps [17,18]. Prior research has shown that such practices can reduce intergroup bias and increase perceived commonality [19,20]. In a recent study, a 10-minute befriending meditation was found to reduce affective polarization in American adults who affiliated with either the Democratic Party or the Republican Party [21]. The study used a pre-posttest design, but it is possible that the repeated administration of affective polarization measures additionally sensitized participants to study hypotheses. The results have also not been replicated in a non-US sample and the psychological mechanisms underlying the effects have yet to be examined.

Here, using a posttest-only randomized experimental design with an online sample of British adults, we investigate whether a brief befriending meditation reduces affective polarization among Remainers and Leavers. Building on prior work, we hypothesized that participants randomized into a befriending condition would score lower on affective polarization than participants randomized into an active control condition. We also made an exploratory hypothesis that the effects would be mediated by perceived commonality between the self and the political outgroup.

## Materials and methods

The study (hypotheses, design plan, sampling plan, variables, and analysis plan) was preregistered on the Open Science Framework (OSF) at https://osf.io/cmzab. The data and the syntax for the analyses can be accessed at https://osf.io/b9762/. Sample size was determined *a priori* using a power analysis (G*Power Version 3.1.9.2). Given that no previous study has investigated the effects of a brief befriending meditation on affective polarization among Remainers and Leavers, we conservatively assumed a small effect size and found that a sample size of 788 participants would achieve 80% power to detect an effect size of d = 0.20 with an alpha of .05 using an independent t-test. We therefore aimed to recruit 400 participants from each Brexit identity category (Remainers and Leavers) and at least 800 in total.

### Participants

Participants were recruited on Prolific Academic (https://app.prolific.co). We used the custom prescreening function to only recruit UK nationals who voted in the Brexit referendum on whether the UK should remain a member of the EU. All participants gave informed consent online and provided relevant information: age (numerical), gender (female, male, other, prefer not to say), having English as first language (yes, no), education level (no formal qualifications, secondary education (e.g. GED/GCSE), high school diploma / A-levels, technical / community college, undergraduate degree (BA/BSc/other), graduate degree (MA/MSc/MPhil/other), doctorate degree (PhD/other), don’t know / not applicable), frequency of meditation practice (never, once per month, two to three times per month, once per week, two to three times per week, four to six times per week, daily), and equipment used to listen to the audio clip (headphones, speakers, other; see S1 File for more information on sample characteristics) through Qualtrics (https://www.qualtrics.com/), the platform used to collect the data for the study. The participants were paid £1.25 for participating the study. The study was approved by the internal review board at NUS.

Stratified random sampling was used to ensure we had approximately equal numbers of Remainers and Leavers based on voting during the referendum. However, many of the participants no longer identified with the same side of the Brexit debate they had voted for in the referendum, which made it difficult to achieve an equal balance of participants currently identified as Remainers and Leavers. In total, 501 Remainers and 433 Leavers completed the study on February 25 and 26, 2021; 476 of them had voted Remain and 458 had voted Leave in the Brexit referendum. Participants who failed the attention check were removed (n = 6), along with participants who took more than an hour to finish the study (n = 6; the average time to finish the study was approximately 16 minutes). The final number of participants was 922 (594 females, 324 males, 3 other, and 1 who preferred not to provide gender information; 495 Remainers and 427 Leavers; aged 18–77 years: M = 24.21, SD = 13.64).

### Design and procedure

We utilized a posttest-only randomized experimental design to assess the effects of a brief befriending meditation on affective polarization. This design was preferred over a pre- and post-test design due to concerns that repeated administration of our affective polarization measures would additionally sensitize participants to our study hypotheses. We compared affective polarization scores of the participants in the befriending condition with the affective polarization scores of the participants in the control condition.

After giving their consent to partake in the study, the participants were asked to indicate whether they currently affiliated with the Remain or Leave side of the Brexit debate. They also answered five items designed to assess how strongly they identified with that side of the Brexit debate (“When I speak about the […] side, I usually say “we” instead of “they””; “When people criticize the […] side, it feels like a personal insult”; “I have a lot in common with other supporters of the […] side”; “When I meet someone who supports the […] side, I feel connected with this person”; “When people praise the […] side, it makes me feel good” [2]). The responses were rated on a 1- (Strongly disagree) to 5-point (Strongly agree) Likert scale. A total score was computed by summing across all items (α = 0.59).

The participants were then randomly assigned to one of the two conditions (befriending, control). Participants in the befriending condition listened to a 10-minute guided befriending meditation. During the recording, the participants in the befriending condition were first instructed to bring friendship and kindness to themselves by repeating the phrase: “May I be free from suffering, may I be happy and healthy, may I have ease of being.” In the same way, the participants were then instructed to bring friendship and kindness to a loved one, a stranger, a difficult person, and all living beings. Participants in the active control condition listened to a 10-minute audio recording about meditation. During the recording, the participants in the active control condition were educated about mindfulness meditation, the neuroscience of mindfulness, and the evidence to date on mindfulness-based programs. The audio for both conditions were recorded by Professor Mark Williams. The audio recording for the befriending condition was derived from *Mindfulness*: *A Practical Guide to Finding Peace in a Frantic World* [22], while the audio recording for the control condition was a combination of talks by Professor Mark Williams (both audio recordings can be accessed at https://osf.io/b9762/). The survey had a timer programmed to prevent participants from moving on from the audio recording before it was finished.

After listening to the audio recording, the participants were presented with an attention check based on the audio recording to which they had been assigned: befriending condition (“During the audio recording, I was instructed to. . .” 1. generate feelings of kindness toward myself and others 2. memorize numbers and dates 3. stretch my body); control condition (“During the audio recording, I learnt about…” 1. mindfulness and meditation 2. sports and gymnastics 3. politics and law). Participants who gave the wrong answer on these questions (n = 6) were excluded from data analyses.

All participants also completed a manipulation check (“How much did you generate feelings of kindness and good-will toward others during the recording you listened to?”), with responses rated on a 1- (Not at all) to 5-point (Very Much) Likert scale. Participants in the befriending condition were expected to provide higher scores than participants in the control condition.

After the attention and manipulation checks, the participants were assessed on perceived commonality with the political outgroup (i.e., Remainers or Leavers) on a scale from 1 to 7 using a modified version of the Inclusion of the Other in the Self Scale [23] (higher scores mean more perceived commonality; see S1 and S2 Figs in S1 File). The participants were also assessed on affective polarization using the feeling thermometer, which asks respondents to rate on a scale from 0 to 100 how cold or warm they feel toward the political ingroup and the political outgroup [24] (higher scores mean more warm feelings toward the target group).

### Statistical analyses

Participants who failed the attention check (n = 6) and participants who took more than an hour to finish the study (n = 6) were excluded from data analyses. Affective polarization scores were determined by calculating the difference between the participants’ feelings toward their own side and the rival side in the Brexit debate. To test our preregistered hypothesis–that participants randomized into a befriending condition would score lower on affective polarization than participants randomized into an active control group–we performed an independent samples t-test to assess whether there was a significant difference in affective polarization scores across the two conditions. As specified in our preregistration, we also performed an exploratory mediation analysis using Hayes’ (2013) SPSS macro (Model 4) with 5,000 bootstrapped estimates [25] to assess whether perceived commonality with the political outgroup mediated the effects of the befriending meditation on affective polarization.

## Results

### Characteristics across conditions

Even though the participants were randomly assigned to one of the two conditions, we first checked that the groups were balanced on characteristics that may affect the results. Independent samples t-tests revealed that there were no significant differences across conditions in age (t(921) = -0.96, p = .337), strength of the Brexit identity (t(921) = 1.07, p = .285), or average length of study completion time (t(921) = 1.60, p = .110). Chi-square tests showed that there were no significant differences across conditions in Brexit identity (χ^2^(2, N = 922) = 0.14, p = .712), gender (χ^2^ (4, N = 922) = 1.43, p = .700), having English as first language (χ^2^ (2, N = 922) = 1.16, p = .281), education level (χ^2^ (12, N = 922) = 11.47, p = .119), frequency of meditation practice (χ^2^ (12, N = 922) = 3.01, p = .807), the equipment used to listen to the audio clip (χ^2^ (4, N = 922) = 2.91, p = .234), or data exclusions (χ^2^ (2, N = 934) = 1.46, p = .227).

### Confirmatory analyses

#### Manipulation check

As a manipulation check, we assessed whether the befriending meditation induced feelings of kindness and good-will toward others more than the control task. An independent samples t-test revealed that there was a significant difference between conditions in feelings of kindness and good-will toward others (t(921) = -3.90, p < .001, d = 0.26), with participants in the befriending condition reporting more kindness and good-will toward others (M = 3.40, SD = 1.00) than participants in the control condition (M = 3.13, SD = 1.12).

#### Affective polarization

We then assessed whether the post-intervention scores of affective polarization (computed from the feeling thermometer) varied between the conditions. An independent samples t-test revealed that there was a small but significant difference between conditions in affective polarization (t(921) = 2.17, p = .030, d = 0.14), with participants in the befriending condition scoring lower on affective polarization (M = 29.26, SD = 29.35) than participants in the control condition (M = 33.43, SD = 28.98; see Table 1 for descriptive statistics). As specified in our preregistration, we also performed a subsequent sensitivity analysis excluding respondents who had affective polarization scores three or more standard deviations from the mean (n = 1), but the result was largely unchanged (t(920) = 2.08, p = .038, d = 0.14).

**Table 1:** Affective polarization scores: Descriptive statistics.

	Remainers	Leavers	Total
Control	40.85 (29.44)	24.62 (25.86)	33.43 (28.98)
Befriending	34.44 (27.43)	23.40 (30.40)	29.26 (29.35)

Mean (standard deviation) reported. Lower scores indicate less affective polarization.

### Exploratory analyses

#### Perceived commonality with the political outgroup

To assess whether perceived commonality with the political outgroup varied between the conditions, we performed an independent samples t-test. The results revealed that there was a small but significant difference between conditions in perceived commonality with the political outgroup (t(921) = -3.54, p < .001, d = 0.23), with participants in the befriending condition scoring higher on perceived commonality with the political outgroup (M = 3.78, SD = 1.71) than participants in the control condition (M = 3.39, SD = 1.62; see Table 2 for descriptive statistics). None of the respondents had perceived commonality scores three or more standard deviations from the mean.

**Table 2:** Perceived commonality scores: Descriptive statistics.

	Remainers	Leavers	Total
Control	3.12 (1.55)	3.72 (1.64)	3.39 (1.62)
Befriending	3.61 (1.57)	3.97 (1.83)	3.78 (1.71)

Mean (standard deviation) reported. Higher scores indicate more perceived commonality.

**Fig 1:**
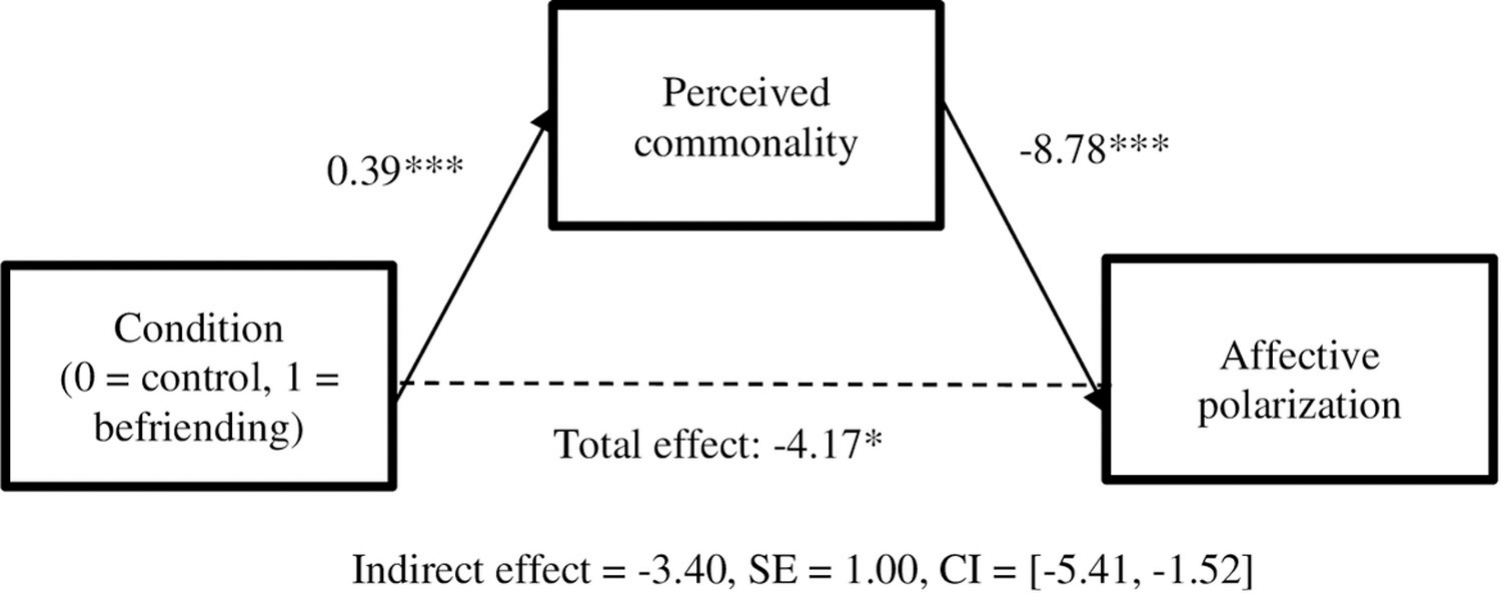
Mediation analysis.

As specified in our preregistration, we then performed an exploratory mediation analysis using Hayes’ (2013) SPSS macros (Model 4) with 5,000 bootstrapped estimates [25] to examine whether perceived commonality with the political outgroup mediated the effects of the meditation intervention (treatment vs. control) on affective polarization (see S2 File for moderated mediation analysis). Results indicated that the indirect effect was significant (b = -3.40, SE = 1.00, bias-corrected 95% CI: [-5.41, -1.52]). After we controlled for perceived commonality with the political outgroup, the direct effect of the befriending meditation on affective polarization was no longer significant (b = -0.77, p = 0.645), suggesting full mediation (see Fig 1).

### Interaction effects

The results suggest that a brief befriending meditation reduces affective polarization between Remainers and Leavers on average. However, it is possible that this effect is moderated by political group. In an additional exploratory analysis, we investigated whether the effects of a brief befriending meditation varied depending on the Brexit identity.

We entered the affective polarization scores into a 2 (condition: befriending, control) x 2 (Brexit identity: Remainers, Leavers) analysis of variance (ANOVA). The results revealed a main effect of the condition (F(1,921) = 4.14, p = .042, η_p_^2^ = .004), a main effect of the Brexit identity (F(1,921) = 52.94, p < .001, η_p_^2^ = .055), but no interaction between the condition and the Brexit identity (F(2,920) = 1.92, p = .166, η_p_^2^ = .002). This suggests that Brexit identity does not influence the effects of a brief befriending meditation on affective polarization.

## Discussion

The results in the present study show that participants in the befriending condition scored modestly lower on affective polarization than participants in the attentional control condition, with the effects mediated by perceived commonality between the self and the political outgroup. The study replicates previous findings using a US sample demonstrating a causal relationship between befriending meditation and affective polarization [21] The mediation effect in this study also helps shed light on a mechanism that may underlie affective polarization that can be targeted in various interventions (e.g., public health campaigns emphasizing commonality across political divisions).

The effect sizes in the present study were small, so it is reasonable to question whether they have practical significance. Of course, even small effects can be important, particularly when a minimal intervention is involved [26]. Given that a recent study found that eight weeks of mindfulness training reduced affective polarization among Remainers and Leavers [27], future studies should examine a larger or potentially more potent dosage of befriending meditation practice (e.g., repeated practice sessions, live instruction). Moreover, the fact that even small effects were detected from a recorded meditation practice supports the possibility of delivery at scale. Meditation-based smartphone apps are far and away the most popular mental health apps [28] and have become the most common way of learning how to meditate [17]. The fact that results did not differ by Brexit identity suggest this may not be a barrier to meditation practice, supporting the potential viability of meditation as a scalable intervention to reduce affective polarization in the UK. Future studies could examine effects of existing meditation-based apps that include similar practices to befriending meditation (e.g., Headspace) on measures of affective polarization.

There are several limitations inherent in the study design. First, all the included measures relied on self-report which are vulnerable to social desirability bias. Second, only the immediate effects of the 10-minute befriending meditation were assessed. Future studies on meditation and affective polarization should include behavioral measures and investigate the short- and long-term effects of meditation practice. Third, there was no pre-test affective polarization measure. Although the groups did not differ on any measured variables at baseline, it is still possible that differences in affective polarization observed at post-test were due to pre-test differences. Another potentially rich future direction could employ qualitative research methods (e.g., in-depth interviews or content analysis of Twitter) to evaluate how meditation training may have impacted affective polarization among British politicians [29].

The findings in this study suggest that a brief befriending meditation can very modestly decrease affective polarization between Remainers and Leavers, with the effects mediated by perceived commonality between the self and the political outgroup. The results build on previous findings and provide additional support for the potential benefits of meditation in political contexts. In sum, audio-guided befriending practices may be a highly scalable means to reduce currently high levels of affective polarization through increasing perceived commonality.

## Supporting information

S1 File Tables and figures. (DOCX)

S2 File Moderated mediation analysis. (DOCX)

S1 Data (ZIP)
