# Supplementary material for: Bridging the (Brexit) divide: Effects of a brief befriending meditation on affective polarization
Source: PLoS One. 2022 May 11;17(5):e0267493. doi: 10.1371/journal.pone.0267493 (PMC9094531; doi:10.1371/journal.pone.0267493)
Supplement: S1 File — (DOCX) [file pone.0267493.s001.docx]

**Supporting Information 1: Tables and Figures**

**Demographic Information**

Participants reported:

Brexit identity (frequencies reported in main text).

Gender and age (descriptive statistics reported in main text).

Whether English was their first language: 98% said “yes”, “2%” said no.

**Table S1. Highest level of education completed.**

| Education Level | Percentage of sample |
| --- | --- |
| No formal qualifications | 0.33% |
| Secondary education (e.g. GED/GCSE) | 12.69% |
| High school diploma / A-levels | 12.91% |
| Technical/community college | 12.47% |
| Undergraduate degree (BA/BSc/other) | 40.78% |
| Graduate degree (MA/MSc/MPhil/other) | 18.33% |
| Doctorate degree (PhD/other) | 2.39% |
| Don't know / not applicable | 0.11% |

**Table S2. Previous meditation experience.**

| Frequency of meditation practice | Percentage of sample |
| --- | --- |
| Daily | 2.93% |
| Four to six times per week | 3.04% |
| Two to three times per week | 6.62% |
| Once per week | 5.21% |
| Two to three times per month | 8.57% |
| Once per month | 14.75% |
| Never | 58.89% |

**Table S3.** **Equipment used to listen to the audio clip (i.e. the intervention).**

| Equipment | Percentage of sample |
| --- | --- |
| Headphones | 65.40% |
| Speakers | 33.95% |
| Other | 0.65% |

**S1 Fig. Perceived commonality with the political outgroup (presented to Leavers).**


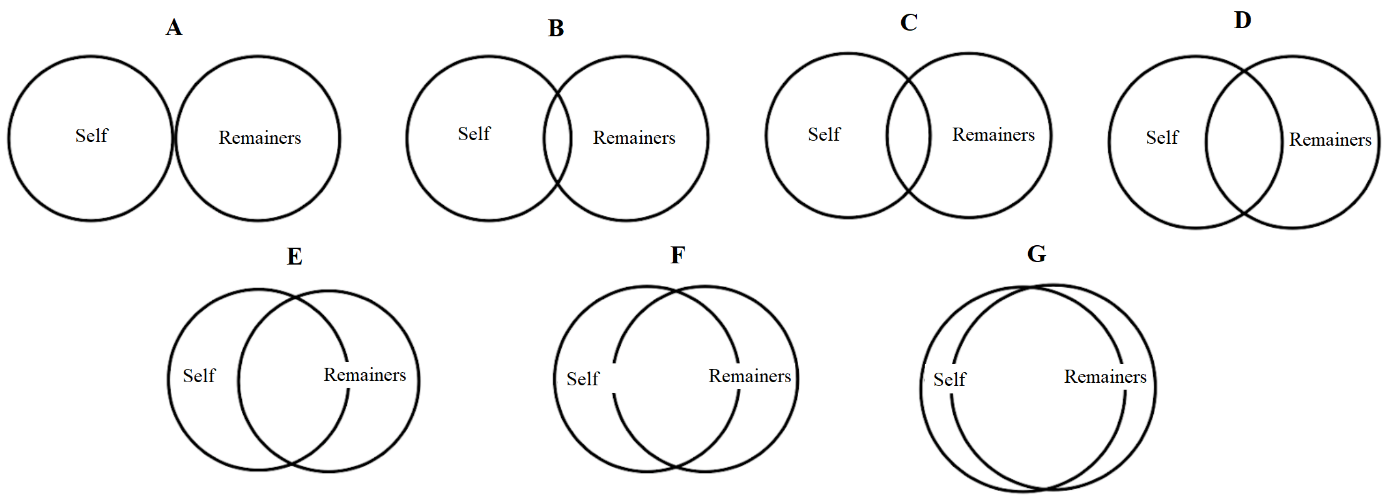


**S2 Fig. Perceived commonality with the political outgroup (presented to Remainers).**

**
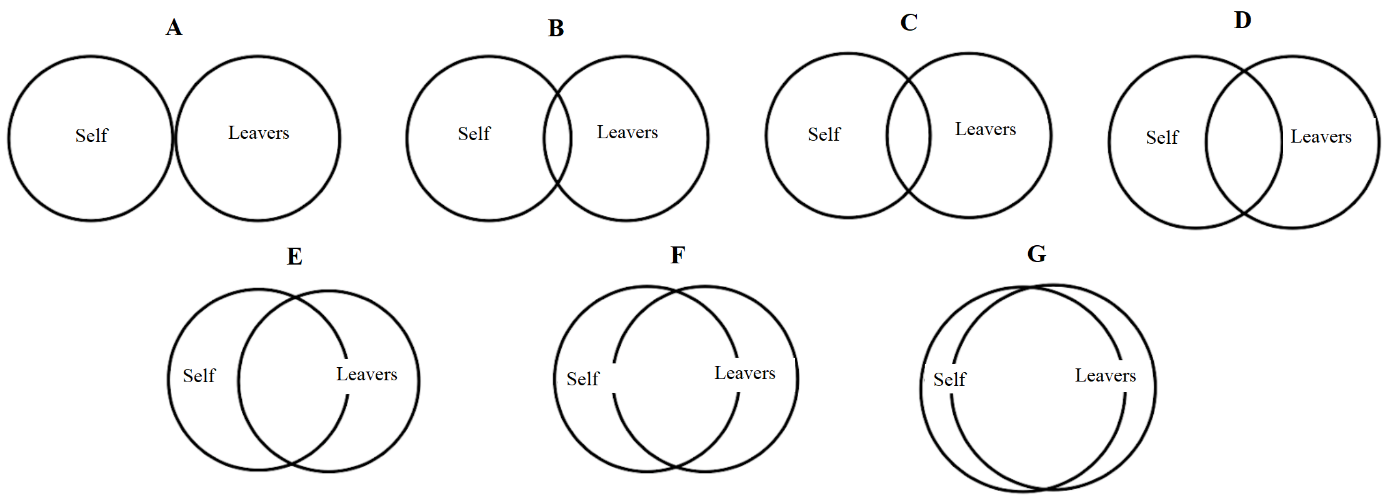
**
