# Supplementary material for: Bridging the (Brexit) divide: Effects of a brief befriending meditation on affective polarization
Source: PLoS One. 2022 May 11;17(5):e0267493. doi: 10.1371/journal.pone.0267493 (PMC9094531; doi:10.1371/journal.pone.0267493)
Supplement: S2 File — (DOCX) [file pone.0267493.s002.docx]

**Supporting Information 2: Moderated Mediation Analysis**

We also ran a moderated mediation analysis using SPSS Process macros (Model 7) with 5,000 bootstrapping resamples to test whether Brexit identity would alter the relationship between treatment and perceived commonality. We positioned treatment as the independent variable, perceived commonality as the mediator, affective polarization as the dependent variable, and Brexit identity as the first-stage moderator. We did not find a significant interaction effect between treatment and Brexit identity (b = .25, SE = .22, p = .25) on perceived commonality. Furthermore, the overall moderated mediation index was not significant (index = -2.18, SE = 1.96, 95%CI [-6.24, 1.52]), indicating that the indirect effect of befriending meditation on affective polarization (through perceived commonality) does not differ in strength based on the participant’s Brexit identity.
